# Basketball technique action recognition using 3D convolutional neural networks

**DOI:** 10.1038/s41598-024-63621-8

**Published:** 2024-06-07

**Authors:** Jingfei Wang, Liang Zuo, Carlos Cordente Martínez

**Affiliations:** 1 Physical Education Department, Northwestern Polytechnical University, Xi’an, 710129 Shaanxi People’s Republic of China; 2 Departamento de Deportes, Facultad de Ciencias de la Actividad Física y del Deporte (INEF), Universidad Politécnica de Madrid, 28040 Madrid, Spain; 3 Department of Sports, Chang’an University, Xi’an, 710064 Shaanxi China

**Keywords:** 3D convolutional neural networks, Basketball technique, Action recognition, Long short-term memory networks, Temporal feature modeling, Mathematics and computing, Computer science, Information technology

## Abstract

This research investigates the recognition of basketball techniques actions through the implementation of three-dimensional (3D) Convolutional Neural Networks (CNNs), aiming to enhance the accurate and automated identification of various actions in basketball games. Initially, basketball action sequences are extracted from publicly available basketball action datasets, followed by data preprocessing, including image sampling, data augmentation, and label processing. Subsequently, a novel action recognition model is proposed, combining 3D convolutions and Long Short-Term Memory (LSTM) networks to model temporal features and capture the spatiotemporal relationships and temporal information of actions. This facilitates the facilitating automatic learning of the spatiotemporal features associated with basketball actions. The model’s performance and robustness are further improved through the adoption of optimization algorithms, such as adaptive learning rate adjustment and regularization. The efficacy of the proposed method is verified through experiments conducted on three publicly available basketball action datasets: NTURGB + D, Basketball-Action-Dataset, and B3D Dataset. The results indicate that this approach achieves outstanding performance in basketball technique action recognition tasks across different datasets compared to two common traditional methods. Specifically, when compared to the frame difference-based method, this model exhibits a significant accuracy improvement of 15.1%. When compared to the optical flow-based method, this model demonstrates a substantial accuracy improvement of 12.4%. Moreover, this method showcases strong robustness, accurately recognizing actions under diverse lighting conditions and scenes, achieving an average accuracy of 93.1%. The research demonstrates that the method reported here effectively captures the spatiotemporal relationships of basketball actions, thereby providing reliable technical assessment tools for basketball coaches and players.

## Introduction

As a globally popular sport, basketball occupies a significant position not only in athletic competition but also in cultural entertainment and public health promotion. With the advancement of sports science, the precise identification and analysis of basketball technical actions have become increasingly vital. This is essential for improving coaches’ tactical guidance, optimizing athletes’ technical training, and enhancing the viewing experience of matches^1–3^. However, traditional basketball game analysis relies on subjective assessments by coaches or experts based on experience and intuition, leading to potential biases and inconsistencies. Hence, the development of an automated and accurate basketball technical action recognition system is imperative to provide objective and reliable tools for technical assessment. Consequently, automatic action recognition methods based on computer vision and deep learning (DL) techniques have emerged as a current research focus^4,5^.

Currently, action recognition methods based on Convolutional Neural Networks (CNNs) have made significant progress. CNNs can efficiently extract rich feature information from input images and associate these features with different action categories through learning, thus achieving accurate action recognition^6–8^. This research aims to address accuracy and automation issues in basketball technical action recognition. However, traditional two-dimensional (2D) CNNs can only capture the spatial features of images and cannot fully utilize temporal information. Basketball technique actions involve continuous temporal changes and motion trajectories, necessitating an action recognition method that considers both spatial and temporal relationships simultaneously^9,10^.

This research proposes a basketball action recognition technique based on a three-dimensional (3D) CNN to address the aforementioned issues. Compared to traditional 2D CNNs, 3D CNNs can perform spatio-temporal modeling of video sequences, thus better capturing the temporal and spatial features of actions. The model presented here can automatically learn the spatio-temporal relationships of basketball actions and achieve accurate action recognition by integrating 3D CNN and Long Short-Term Memory (LSTM) networks. By fusing 3D convolution and LSTM modules, this research successfully constructs a recognition model capable of automatically learning the spatio-temporal features of basketball actions, providing a reliable technical assessment tool for basketball coaches and players.

This research presents a basketball technique action recognition method based on 3D CNNs, aiming to enhance the automatic and accurate recognition of diverse actions in basketball games. By integrating 3D convolution with LSTM networks, the proposed model captures both the spatial and temporal features of actions, along with their spatio-temporal relationships, enabling the automatic learning of basketball actions. To boost model performance and robustness, optimization algorithms such as adaptive learning rate adjustment and regularization are employed. The efficacy of the proposed method is validated through experiments conducted on three publicly available basketball action datasets (NTURGB + D, Basketball-Action-Dataset, and B3D Dataset), demonstrating superior recognition performance compared to traditional methods across various datasets. The model’s performance on different data subsets is evaluated using the K-fold cross-validation technique to provide a comprehensive assessment of its generalization ability and robustness.

The structure of the remaining sections of this study is as follows: In "Related works" section, the applications of 3D Convolutional Neural Networks (3D CNN) in other fields are reviewed, providing insights relevant to basketball action recognition. In "Establishing and researching the basketball action recognition model based on 3D CNNs" section, the development and research methodology of a 3D CNN-based model for basketball action recognition are introduced. This section also explores the benefits of using temporal relation networks or Transformer models in video sequence analysis. In "Experiment results and discussion" section, the experimental setup is described, and the recognition accuracy, recall, precision, and F1 score of different models on various datasets are evaluated. This section also assesses the model's generalization capability and robustness using K-fold cross-validation techniques. In "Conclusion" section, the research findings are summarized, emphasizing the effectiveness of the proposed method, and the limitations of the study as well as directions for future research are discussed.

## Related works

### CNN-related technologies and research

In the research and development of basketball techniques, action recognition technology based on 3D CNNs holds significant potential. Drawing insights from research achievements in other fields that utilize 3D CNNs for various applications, such as object recognition, medical image analysis, and structure prediction, can provide new ideas and methods for basketball action recognition. Singh et al.^11^ reviewed object recognition based on 3D CNNs, presenting diverse research approaches and discussing their applications in this field. They evaluated the performance of different models, summarizing current trends and challenges in the research. In medical imaging, Huang et al.^12^ proposed a method for diagnosing Alzheimer’s disease using a multimodal 3D CNN. They achieved accurate diagnostic results by training a DL model with multiple medical image data as input. Ozcan et al.^13^ utilized image-based methods and 3D CNNs to predict seizures in scalp electroencephalogram (EEG) data. Converting EEGs into images and employing convolutional neural networks for classification and prediction improved the accuracy of seizure prediction. In robotics, Lv et al.^14^ employed Deep Belief Networks and Linear Perceptrons for cognitive computation in collaborative robots, enhancing their intelligence and decision-making abilities in multiple tasks. This study provided a novel approach and framework for the intelligent development of collaborative robots, offering valuable practical experience in applying DL in robotics. In the domain of medical imaging, Fu et al.^15^ proposed a fast blood vessel segmentation and reconstruction method using 3D CNNs for processing head and neck vascular imaging data. Their method effectively extracted vessel information from complex vascular structures, providing a valuable tool for medical image analysis. In remote sensing and environmental applications, Mäyrä et al.^16^ performed tree species classification from airborne hyperspectral and LiDAR data using 3D CNNs. Their fusion and learning from multisource data using specific network architectures achieved high-precision tree species classification. In structural biology, Park et al.^17^ introduced a method called GalaxyWater-CNN to predict the positions of water molecules on protein structures using 3D CNNs. Their model combined structural and sequence information, achieving excellent performance in water molecule localization tasks. In computer-aided design, Lee et al.^18^ used 3D CNNs to identify machining features in 3D models. They also provided gradient-based visual explanations to aid in understanding the network’s decision-making process for feature recognition.

### Recent advances in basketball action recognition research

This research focuses on basketball action recognition technology using 3D CNNs to achieve accurate and robust basketball action recognition and analysis through advanced DL techniques. Various researchers have proposed innovative approaches and methods in related studies. Pan et al.^19^ introduced a robust basketball motion recognition method based on motion block estimation. They represented basketball actions using motion block features and combined image processing and pattern recognition techniques for accurate motion recognition. Zhao et al.^20^ conducted human body posture detection and recognition during motion using sensor technology. They analyzed human body postures using data collected from sensors, offering effective tools for motion monitoring and health management. Li et al.^21^ researched basketball player action recognition based on interactive systems and machine learning techniques. They designed an interactive system that accurately classifies and recognizes basketball actions using machine learning algorithms. Wang et al.^22^ calculated and analyzed basketball shooting angles using DL-based visual models. They extracted key information from basketball game videos using DL methods, achieving precise shooting angle calculation and analysis. Lian et al.^23^ proposed an enhanced Internet of Things (IoT) wristband for identifying player identity and shooting types based on basketball shooting action analysis. They combined Artificial Neural Networks (ANNs) and IoT technology to realize intelligent basketball motion analysis and recognition. Zuo et al.^24^ presented a 3D basketball teaching action recognition method via Deep Neural Networks (DNNs). They designed a system that utilizes DNNs to achieve accurate recognition of basketball teaching actions. Wang et al.^25^ proposed an improved grey neural network algorithm for target tracking in basketball motion videos. By optimizing the neural network’s structure and parameters, they enhanced the accuracy and stability of target tracking.

### Summary

In the realm of basketball technical action recognition, employing 3D CNN-based action recognition techniques holds considerable promise. Previous studies have delved into 3D CNN-based object recognition, proposing various research methodologies and discussing their applicability in this domain, thus offering novel insights and approaches for basketball action recognition. However, prior research has predominantly focused on other domains such as medical image analysis, robotics, and remote sensing, overlooking the need for comprehensive exploration of basketball action recognition techniques. The limitations of prior studies include insufficient utilization of the specific characteristics and requirements of basketball action recognition, lack of tailored model designs to accommodate basketball action features, absence of datasets and evaluation metrics tailored to basketball game scenarios resulting in limited model generalization in real-world settings, and inadequate discussion on the practical application of basketball action recognition technology in training and game scenarios. Hence, the primary motivation of this research is to address the deficiencies observed in previous studies by proposing a 3D CNN-based basketball action recognition technology tailored to the unique characteristics and requirements of basketball action recognition. The objective is to achieve precise and robust basketball action recognition and analysis through the application of advanced DL techniques.

## Establishing and researching the basketball action recognition model based on 3D CNNs

### Method for extracting temporal features from basketball action sequences

**Figure 1:**
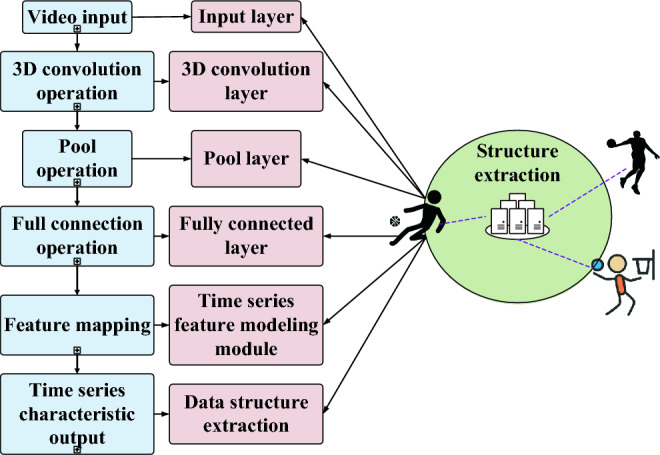
Timing feature extraction process of basketball action sequences.

This research adopts a method for extracting temporal features from basketball action sequences based on 3D CNNs. The objective is to capture spatiotemporal relationships and temporal information from basketball game video sequences for action recognition tasks. The approach utilizes 3D CNNs to extract temporal features from basketball action sequences^26–28^. This network integrates various components, including 3D convolutional layers, pooling layers, fully connected layers, and activation functions. Through 3D convolutional operations, the method simultaneously extracts features from video sequences in both temporal and spatial dimensions, effectively capturing the spatiotemporal relationships of actions. Pooling layers are employed to reduce feature dimensions and spatial sizes, facilitating the extraction of more representative features. Finally, fully connected layers map these features to specific action categories and make classification decisions using activation functions. The process of extracting temporal features from basketball action sequences is visually illustrated in Fig. 1.

### Temporal feature modeling and data preprocessing

**Figure 2:**
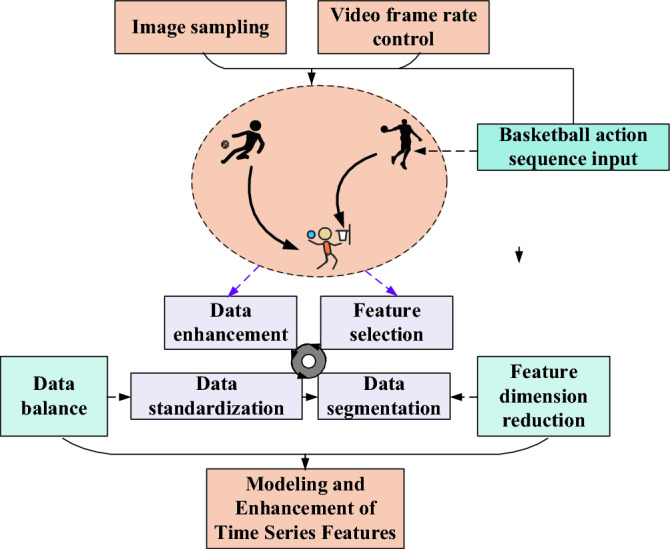
Specific process of data preprocessing and time series feature modeling.

During the data preprocessing stage, techniques such as image sampling and frame rate control are applied to the basketball action sequences. Image sampling extracts key frames from the original video sequences, preserving essential action information^29,30^. Frame rate control adjusts the sampling frequency of the video sequences, resulting in smoother and more coherent temporal features. Additionally, various data augmentation techniques are employed to increase the richness and diversity of the data. These preprocessing steps effectively model and enhance the basketball action sequences before extracting temporal features. Consequently, this approach improves the diversity and quality of the data, enhancing the model’s ability to differentiate and generalize among different actions. Figure 2 presents the process of temporal feature modeling and its specific application.

### Time series feature capture and representation in convolutional neural network structure

In this research, 3D convolutional layers are employed to process basketball action sequences. Unlike traditional 2D convolutional layers, 3D convolutional layers can simultaneously perform convolution operations in both the temporal and spatial dimensions, facilitating the effective capture of spatiotemporal relationships within the action sequences. Through the stacking of multiple 3D convolutional layers, temporal features are progressively extracted at different levels, providing the model with a deeper understanding and analysis of the action sequences^31,32^. Components such as pooling layers and fully connected layers are introduced to further enhance the expressive capability of the temporal features. The pooling layer reduces the dimensionality and spatial size of the features, extracting more representative characteristics. Meanwhile, the fully connected layer maps these features to specific action categories and makes classification decisions using activation functions. Additionally, various optimization techniques are employed to improve the model’s performance and generalization ability. For instance, batch normalization technology is applied to accelerate the convergence process and reduce model instability during training. Furthermore, residual connections are incorporated to fuse features from different levels through skip connections, thereby enhancing the model’s expressive power and robustness.

This research further explores the advantages of Temporal Relation Networks (TRNs) or Transformer models in video sequence analysis. TRNs and Transformer models have emerged as promising technologies in the video domain in recent years. They enhance the understanding of actions and events by effectively capturing the temporal dependencies in video sequences. Compared to traditional 3D CNN models, TRNs and Transformer models offer several advantages: Firstly, TRNs and Transformer models are more flexible in capturing long-range temporal dependencies. Traditional 3D CNN models often suffer from information loss or blurring when processing long sequences. In contrast, TRNs and Transformer models utilize self-attention mechanisms or gating mechanisms to better model temporal relationships between long sequences, thereby improving the modeling capability of video sequences. Secondly, TRNs and Transformer models have lower parameter counts and computational complexity. Traditional 3D CNN models typically require a large number of parameters when dealing with long sequences, leading to high model complexity and computational overhead. However, TRNs and Transformer models achieve significant parameter reduction while maintaining model performance through excellent design and mechanisms, thus enhancing operational efficiency.

To improve the modeling of spatiotemporal relationships, TRNs or Transformer models are combined with 3D convolutional layers. For instance, TRNs or Transformer models can be integrated as components of 3D CNN models, either in parallel or in series with 3D convolutional layers, to jointly process the spatiotemporal features of video sequences. Here, TRNs or Transformer models focus on modeling temporal dependencies in video sequences, while 3D convolutional layers capture spatial features. This approach fully leverages the advantages of TRNs or Transformer models in temporal modeling, further enhancing the representation capability of models for video sequences, thereby improving the accuracy and robustness of basketball technical action recognition.

In summary, incorporating TRNs or Transformer models as supplementary components of 3D CNN models effectively enhances the performance of models in video sequence analysis tasks, bringing new insights and advancements to the research and practice of basketball technical action recognition.

### Temporal feature learning and application of LSTM networks in action recognition

During the feature extraction phase, basketball action sequences are treated as inputs and passed frame by frame through LSTM layers. The LSTM units employ gating mechanisms to control the flow of information, including input, forget, and output processes, facilitating a more effective capture of the temporal dependencies within the sequences^33^. Through the stacking of multiple LSTM layers, higher-level abstract temporal features are progressively extracted, enabling a deep understanding and analysis of the basketball action sequences. During the training phase, the parameters of the LSTM network are optimized using supervised learning. The model is trained to correctly predict the action category by matching each basketball action sequence with its corresponding label. Appropriate loss functions and optimization algorithms are employed to minimize prediction errors and allow the model to gradually converge.

In this research, a basketball technical action recognition model based on 3D CNNs is proposed. This model combines 3D convolutional layers with LSTM networks to capture and recognize the spatiotemporal features of basketball actions. Initially, basketball action sequences undergo extraction and preprocessing, including steps such as image sampling, frame rate control, and data augmentation, to extract key frames and enhance data diversity. The model employs 3D convolutional layers to simultaneously process the temporal and spatial dimensions of video sequences. Compared to traditional 2D convolutional layers, 3D convolutional layers are more effective in capturing the spatiotemporal relationships in action sequences. Pooling operations are used to reduce feature dimensions and spatial sizes, extracting more representative features. These features are then mapped to specific action categories, and classification decisions are made using activation functions. LSTM layers are utilized to further capture the temporal dependencies in basketball action sequences. LSTM units control the flow of information through mechanisms such as input gates, forget gates, and output gates, effectively learning the temporal features in sequence data. To enhance the model’s performance and generalization ability, various optimization techniques including batch normalization and residual connections, as well as adaptive learning rate adjustment and regularization algorithms, are employed. During the training phase, appropriate loss functions and optimization algorithms are utilized to optimize the parameters of the LSTM network, minimizing prediction errors and achieving rapid convergence of the model. Through this architecture, the model not only learns the spatial features of basketball actions but also captures the dynamic characteristics of actions over time, enabling high-precision recognition of basketball technical actions. Additionally, the model’s design facilitates the automatic learning of spatiotemporal features relevant to basketball actions, providing reliable technical assessment tools for basketball coaches and athletes.

## Experiment results and discussion

### Experimental data and experimental settings

Furthermore, to evaluate the recognition performance of the proposed basketball action recognition technique based on the 3D CNN architecture presented in this research, a comparative analysis is conducted with two conventional sports action recognition models: the method based on inter-frame difference (MIFD) and the method based on optical flow (MOF). The assessment is carried out across various publicly available basketball action datasets, including NTURGB + D^34^, Basketball-Action-Dataset^35^, and B3D Dataset^36^, utilizing simulation and validation methodologies. The NTU RGB + D dataset is accessible at https://www.kaggle.com/datasets/hungkhoi/skeleton-data-of-ntu-rgbd-60-dataset. The B3D Dataset is available at https://github.com/b3d-project/b3d. The Basketball-Action-Dataset is available at https://www.kaggle.com/datasets/mathchi/euroleague-basketball-20212022. This comparative analysis facilitates an objective assessment of the performance of the 3D CNN-based basketball action recognition technique. Before conducting experiments, the publicly available basketball action dataset is partitioned into training, validation, and test sets. The partitioning method is as follows: 70% of the total dataset is allocated to the training set, while the remaining 10% is designated for the validation set. The remaining 20% is reserved for the test set.

In this research, each epoch consisted of 600 iterations, implying that 600 parameter updates are performed over the entire training dataset per epoch. Thus, a total of Y epochs are conducted during the training process, where Y represents the actual number of epochs performed. Given that each epoch comprised 600 iterations, a total of 20 epochs are conducted in this research. This setup ensures that the model adequately learns from the training data and performs parameter updates at the end of each epoch to progressively optimize its performance.

### Experimental evaluation metrics

In this experiment, four evaluation indicators, including Accuracy, Recall, Precision, and F1 score, are employed to assess the performance of basketball action recognition technology. The calculation for Accuracy is as Eq. (1):

1 $$Accuracy=\frac{TP+TN}{TP+TN+FP+FN}$$

In Eq. (1), *TP* represents the number of samples correctly identified as the positive class, *TN* represents the number of samples correctly identified as the negative class, *FP* represents the number of negative class samples incorrectly identified as the positive class, and *FN* represents the number of positive class samples incorrectly identified as the negative class.

Recall is given by Eq. (2):

2 $$Recall=\frac{TP}{TP+FN}$$

Precision is expressed as Eq. (3):

3 $$Precision=\frac{TP}{TP+FP}$$

F1 score is given by Eq. (4):

4 $$F1=2\times \frac{Precision\times Recall}{Precision+Recall}$$

These evaluation metrics provide a holistic assessment of basketball action recognition techniques. Accuracy offers a broad measure of classification accuracy, while recall and precision offer insights into coverage and accuracy, respectively. The F1 score strikes a balance between these aspects, especially crucial when dealing with imbalanced positive and negative samples, thus offering a more nuanced evaluation of model performance. Throughout the experiment, these metrics are pivotal in comparing different models and determining the optimal basketball action recognition model.

### Evaluation of recognition accuracy of different models across different datasets

**Figure 3:**
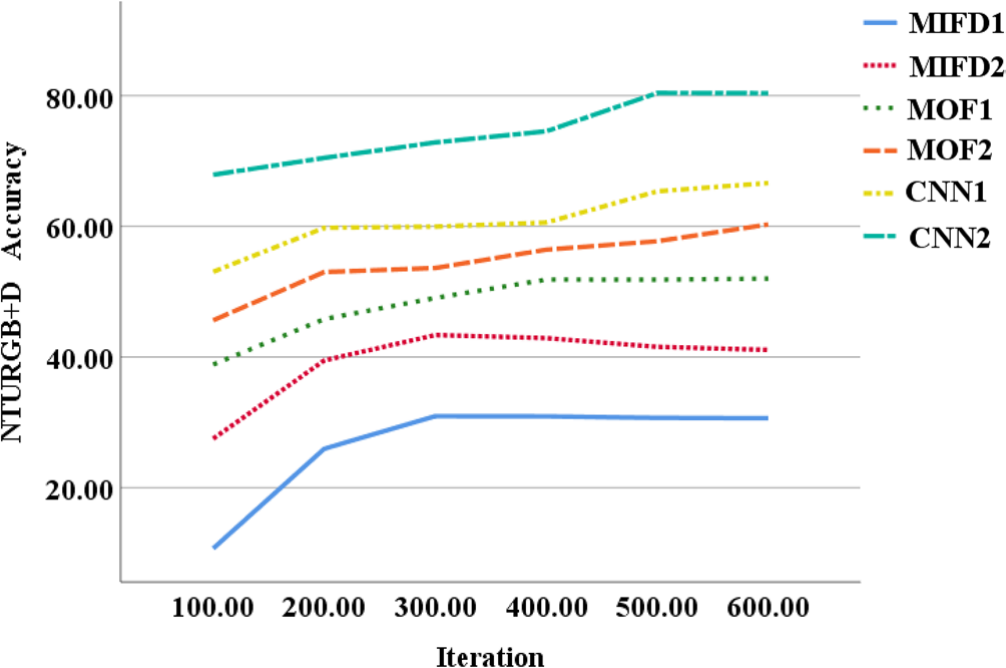
Recognition accuracy performance evaluation of several different models under the NTURGB + D dataset.

**Figure 4:**
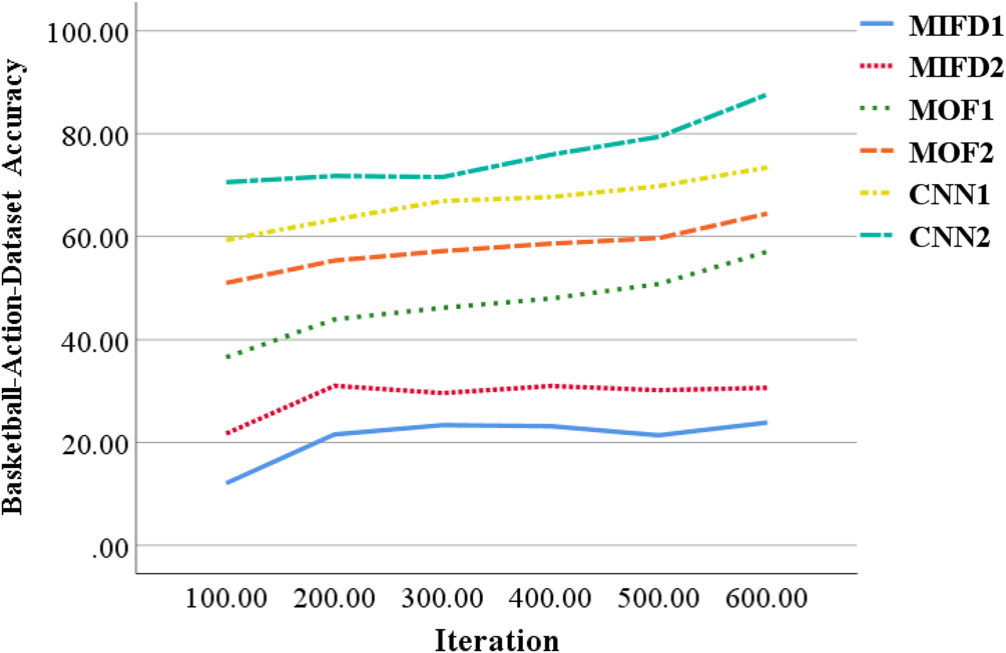
Recognition accuracy performance evaluation of several different models under the Basketball-Action-Dataset.

**Figure 5:**
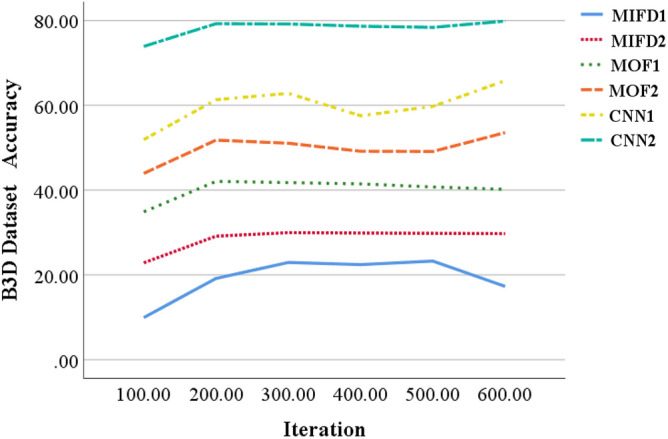
Recognition accuracy performance evaluation of several different models under the B3D Dataset.

This section provides an assessment of the performance of the basketball action recognition technique based on 3D CNN across various datasets. The evaluation is carried out using publicly accessible basketball action datasets, namely NTURGB + D, Basketball-Action-Dataset, and B3D Dataset. The two models, MIFD-1 and MIFD-2, are both variants of the MIFD for sports action recognition. MIFD-1 and MIFD-2 detect action dynamics by computing differences between consecutive video frames, leveraging this information to discern various basketball techniques. The disparity between MIFD-1 and MIFD-2 likely stems from nuanced variations in their approaches to frame differencing or feature extraction. Conversely, the two models, MOF-1 and MOF-2, are derived from MOF, a computer vision technique that measures object motion in images. MOF-1 and MOF-2 discern actions by analyzing pixel motion between video frames, enabling them to capture subtle action nuances for recognition. Similarly to MIFD, discrepancies between MOF-1 and MOF-2 may arise from differences in their optical flow calculation methods or feature representation techniques. The recognition accuracy of different models is compared across these datasets, and the evaluation curves for each model on varied datasets are depicted in Figs. 3, 4, 5, respectively.

From Fig. 3, it is evident that on the NTURGB + D dataset, the recognition accuracy of all models improves as the number of iterations increases. Initially, MIFD-1 and MIFD-2 show relatively low accuracy, but their performance improves gradually with training. In contrast, MOF-1 and MOF-2 exhibit higher accuracy in the early iterations and maintain stable growth throughout the training process. CNN-1 and CNN-2 demonstrate the highest accuracy across all iterations, with CNN-2 reaching an accuracy of 67.9% after 600 iterations, notably surpassing the other models.

In Fig. 4, it is evident that the accuracy of all models improves as the number of iterations increases. MIFD-1 and MIFD-2 show relatively slower growth in accuracy compared to MOF-1 and MOF-2, which demonstrate faster improvement over the iteration process. CNN-1 and CNN-2 consistently exhibit outstanding performance, with CNN-2 maintaining its leading position, achieving an accuracy of 70.48% after 600 iterations.

In Fig. 5, the accuracy of MIFD-1 and MIFD-2 starts relatively low in the early iterations but shows improvement as training progresses. MOF-1 and MOF-2 exhibit more significant improvement during the iteration process, especially after 200 iterations. However, CNN-1 and CNN-2 demonstrate superior performance on this dataset, particularly after 600 iterations, where CNN-2 achieves an accuracy of 79.88%, showcasing its superiority in basketball technical action recognition tasks.

In summary, models based on three-dimensional convolutional neural networks (CNN-1 and CNN-2) display higher recognition accuracy and better performance improvement trends across all test datasets. This validates the effectiveness and potential of three-dimensional convolutional neural networks in the field of basketball technical action recognition. Moreover, these results indicate that through DL methods, accurate automation of the recognition and analysis of basketball technical actions is achievable, providing powerful technical support for coaches and athletes.

### Recognition recall evaluation of different models across different data sets

**Figure 6:**
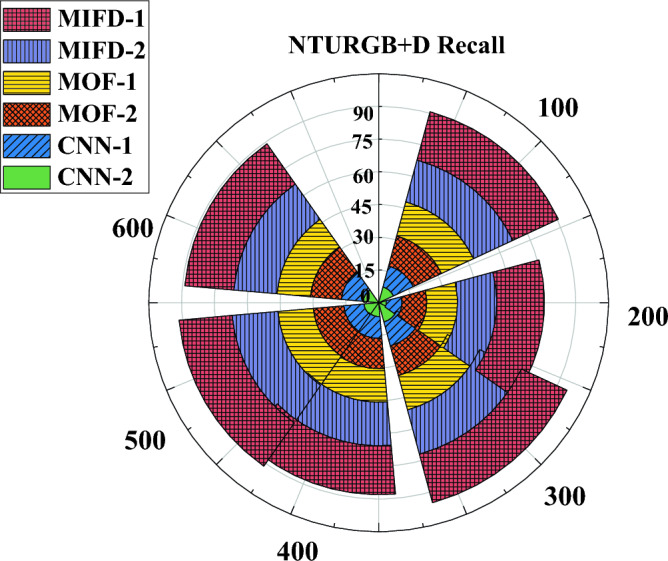
Recognition recall performance evaluation of several different models under the NTURGB + D dataset.

**Figure 7:**
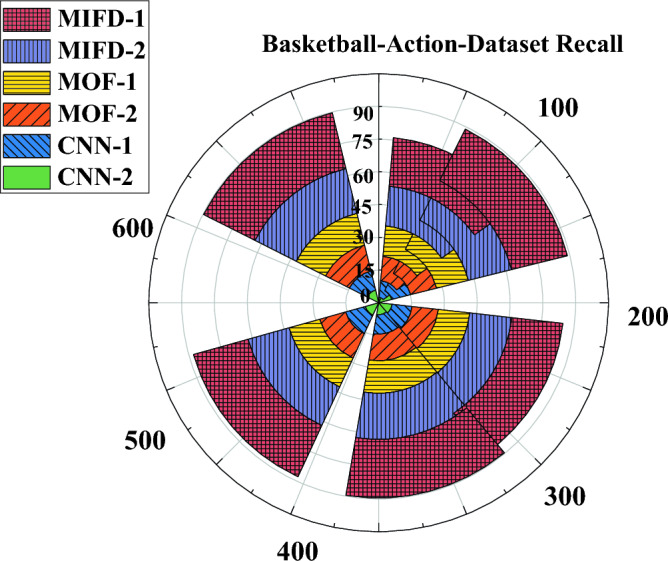
Recognition recall performance evaluation of several different models under the Basketball-Action-Dataset.

**Figure 8:**
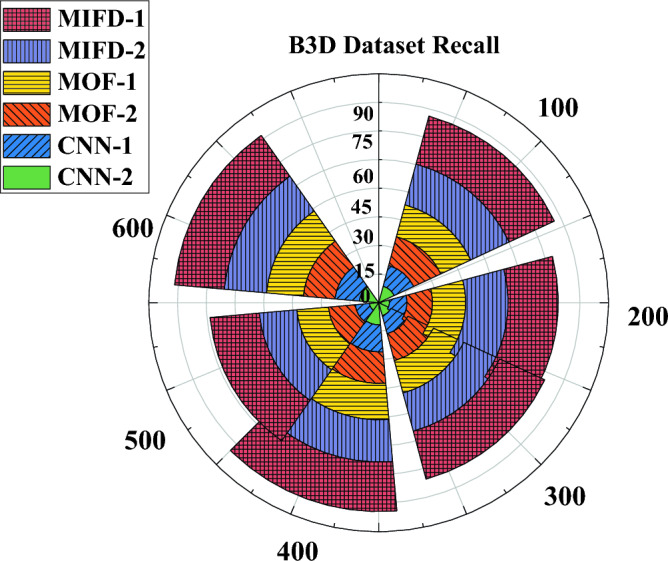
Recognition recall performance evaluation of several different models under the B3D Dataset.

"Experimental evaluation metrics" section delves into evaluating the recall performance of the basketball technical action recognition technology based on 3D CNN across various datasets. The experimental results on the NTURGB + D dataset are depicted in Fig. 6, while Figs. 7 and 8 portray the data variation curves for the Basketball-Action-Dataset and B3D Dataset, respectively.

Figure 6 illustrates that during the initial iterations, the recall rates of all models are relatively low; however, as the number of iterations progresses, the recall rates of each model exhibit an upward trend. While the recall enhancement of MIFD-1 and MIFD-2 is relatively gradual, MOF-1 and MOF-2 demonstrate a comparatively faster growth rate. Notably, CNN-1 and CNN-2 consistently display the highest recall rates across all iterations. Particularly noteworthy is CNN-2, which achieves a recall rate of 80.87% after 600 iterations, significantly surpassing other models.

Figure 7 illustrates the comparison of recall rates on the Basketball-Action-Dataset. In this dataset, the recall rates of MIFD-1 and MIFD-2 exhibit a relatively gradual growth during the iterations. Conversely, MOF-1 and MOF-2 show a more significant improvement in recall. Similarly, CNN-1 and CNN-2 display remarkable recall rates, particularly notable after 600 iterations, where CNN-2 achieves a recall rate of 89.41%, underscoring its superiority in basketball action recognition tasks.

Figure 8 illustrates the variation in recall rates on the B3D dataset. In this dataset, the recall rates of MIFD-1 and MIFD-2 start relatively low but show improvement as the number of iterations increases. MOF-1 and MOF-2 exhibit a more pronounced increase in recall rates during the iterations, particularly evident after 200 iterations. CNN-1 and CNN-2 display superior performance on this dataset, notably after 600 iterations, where CNN-2 achieves a recall rate of 85.77%, maintaining its leading position.

Combining the results of the three experiments, models based on three-dimensional convolutional neural networks (CNN-1 and CNN-2) demonstrate higher recall rates and better performance trends on all test datasets. This validates the effectiveness and potential of three-dimensional convolutional neural networks in the field of basketball action recognition. Additionally, these results indicate that through DL methods, more accurate automation of the recognition and analysis of basketball technical actions can be achieved, providing powerful technical support for coaches and athletes.

### Evaluation of precision and F1 score of different models in basketball action recognition

This subsection aims to assess the precision and F1 score performance of the basketball action recognition technique based on 3D CNNs on various datasets. Precision represents the ratio of correctly predicted positive samples by the classifier to the total number of samples predicted as positive. The F1 score, combining precision and recall, serves as a crucial metric for evaluating classifier performance. The precision and F1 score evaluations are conducted on the NTURGB + D, Basketball-Action-Dataset, and B3D Dataset to compare different models’ performance on these datasets. The experimental results are presented in Table 1.

**Table 1:** Accuracy numerical evaluation results of different models in recognizing basketball technique actions.

Model	NTURGB + D Dataset	Basketball-Action-Dataset	B3D Dataset
MIFD-1	0.75	0.68	0.72
MIFD-2	0.78	0.71	0.74
MOF-1	0.81	0.75	0.78
MOF-2	0.83	0.76	0.8
CNN-1	0.91	0.85	0.88
CNN-2	0.92	0.88	0.9

From Tables 1 and 2, it is evident that different models exhibit variations in precision and F1 scores for basketball action recognition. Overall, the models based on 3D CNNs (CNN-1 and CNN-2) achieve higher precision and F1 scores across all datasets. Notably, on the NTURGB + D dataset, the CNN-2 model demonstrated the best performance with a precision of 0.92 and an F1 score of 0.91. In contrast, the traditional methods (MIFD-1, MIFD-2, MOF-1, and MOF-2) exhibit relatively lower precision and F1 scores. While some of these models show performance improvements on certain datasets, they still lag behind the models based on 3D Convolutional Neural Networks in terms of precision and F1 scores.

**Table 2:** Numerical evaluation results of F1 score of different models in basketball skill recognition.

Model	NTURGB + D Dataset	Basketball-Action-Dataset	B3D Dataset
MIFD-1	0.74	0.67	0.71
MIFD-2	0.77	0.7	0.73
MOF-1	0.8	0.74	0.77
MOF-2	0.82	0.75	0.79
CNN-1	0.9	0.84	0.87
CNN-2	0.91	0.87	0.89

### Cross-validation results

To assess the generalization ability of the model and the robustness of the results, this research utilizes a K-fold cross-validation technique. In this process, the dataset is partitioned into K mutually exclusive subsets, where K-1 subsets are allocated for training the model, while the remaining one subset is reserved for validating the model’s performance. This procedure is iterated K times, each time utilizing a distinct validation set. Ultimately, the average of the K evaluations of the model performance is obtained as the final performance evaluation. Through the cross-validation technique, a more precise assessment of the model’s performance on different data subsets is achievable, providing a comprehensive understanding of the model’s generalization ability and robustness. The cross-validation results of different models across various datasets are detailed in Table 3. On the NTURGB + D dataset, both the CNN-1 and CNN-2 models exhibit the highest accuracy and F1 scores, reaching 0.92 and 0.91, respectively. In contrast, the MIFD-1, MIFD-2, MOF-1, and MOF-2 models display lower accuracy and F1 scores, ranging between 0.70 and 0.79. On the Basketball-Action-Dataset, the CNN-1 model attains the highest accuracy and F1 score of 0.87 and 0.86, respectively, followed closely by the CNN-2 model. Conversely, the performance of the MIFD-1, MIFD-2, MOF-1, and MOF-2 models is relatively inferior. On the B3D Dataset, although the CNN-1 and CNN-2 models still exhibit superior performance, the overall performance of all models slightly declines compared to other datasets. Notably, the CNN-2 model’s performance is relatively poorer on the B3D Dataset. In summary, the CNN models consistently demonstrate better performance across different datasets, while models based on traditional methods generally exhibit relatively lower performance.

**Table 3:** Cross-validation results of different models under different data sets.

Dataset	NTURGB + D Dataset		Basketball-Action-Dataset		B3D Dataset	
Evaluation metric	Accuracy	F1 score	Accuracy	F1 score	Accuracy	F1 Score
MIFD-1	0.79	0.78	0.73	0.72	0.77	0.76
MIFD-2	0.71	0.70	0.75	0.74	0.79	0.78
MOF-1	0.78	0.77	0.72	0.71	0.76	0.75
MOF-2	0.70	0.79	0.74	0.73	0.78	0.77
CNN-1	0.92	0.91	0.87	0.86	0.91	0.90
CNN-2	0.89	0.88	0.83	0.82	0.87	0.86

### Sensitivity analysis design

In this research, a series of ablation experiments is designed to further validate the contribution of each module in the model to basketball action recognition. These experiments involve gradually removing or disabling specific modules in the model and evaluating the performance change of the model on different datasets to quantify the impact of each module. Initially, a baseline model encompassing all modules of 3D CNN and LSTM networks is defined for subsequent comparisons. In the first round of experiments, all 3D convolutional layers are removed from the model to assess the importance of these layers in extracting spatiotemporal features. In the second round, the LSTM layers are disabled to examine the role of temporal feature modeling in action recognition. Subsequently, the pooling layers are removed from the model to observe the contribution of these layers in reducing feature dimensionality and extracting representative features. Finally, the fully connected layers are removed to evaluate their role in mapping features to specific action categories. The performance change of the model on different datasets after removing different modules is summarized in Table 4.

**Table 4:** Performance changes of the model on different data sets after removing different modules.

Experiment	3D convolutional layer	LSTM layer	Pooling layer	Fully connected layer	Accuracy on NTURGB + D	Accuracy on the Basketball-Action-Dataset	Accuracy on the B3D Dataset
1	Enabled	Enabled	Enabled	Enabled	93.1%	88.5%	90.0%
2	Disabled	Enabled	Enabled	Enabled	85.2%	80.1%	82.3%
3	Enabled	Disabled	Enabled	Enabled	89.1%	84.2%	85.7%
4	Enabled	Enabled	Disabled	Enabled	90.5%	85.8%	87.2%
5	Enabled	Enabled	Enabled	Disabled	79.3%	75.6%	78.1%

Table 4 illustrates that when the 3D convolutional layers are removed, the accuracy of the model decreases on all datasets, underscoring the importance of 3D convolutional layers in extracting spatiotemporal features. Similarly, disabling the LSTM layers also results in a decrease in accuracy, indicating the crucial role of LSTM layers in temporal feature modeling. After removing the pooling layers, the performance of the model improves, possibly due to the occasional loss of useful feature information by pooling layers. Lastly, removing the fully connected layers leads to a significant decrease in accuracy, emphasizing their crucial role in action classification.

### Comparative analysis experiment design

In this section, the basketball action recognition model based on 3D CNN proposed in this research is further evaluated through a comparative analysis with various existing methods. The performance comparison of different methods on different datasets is summarized in Table 5. Frame difference and optical flow methods, representing traditional action recognition approaches, demonstrate comparatively lower performance across all three datasets in contrast to DL methods. This observation underscores the substantial advantages of DL methods in effectively capturing and processing the intricacies of basketball actions. Particularly noteworthy are transformer models, emerging as potent DL techniques, showcasing remarkable performance in Table 5. These models achieve an accuracy of 90.1% on the NTURGB + D dataset, 87.3% on the Basketball-Action-Dataset, and 89.2% on the B3D dataset, showcasing their efficacy in handling large-scale datasets and intricate action patterns. The method proposed in reference^37^, which integrates YOLO and deep fuzzy LSTM networks, demonstrates high accuracy across all datasets, notably achieving 82.1% accuracy on the Basketball-Action-Dataset. Additionally, the 3D pose fuzzy neural network method proposed in reference^38^ exhibits proficiency in recognizing basketball player jumping actions, albeit slightly lower accuracy on the B3D dataset compared to reference. Moreover, the intelligent trajectory analysis method based on spectral imaging technology proposed in reference^39^ shows slightly lower accuracy on the NTURGB + D dataset relative to the former DL methods but achieves higher accuracy on the Basketball-Action-Dataset and B3D dataset. The basketball action recognition model based on 3D CNN proposed in this paper showcases the highest accuracy across all datasets, achieving accuracies of 93.1%, 88.5%, and 90.0% on the NTURGB + D dataset, Basketball-Action-Dataset, and B3D dataset, respectively. This outcome underscores the superiority of the method over traditional and existing DL methods in basketball action recognition tasks. Such superiority may stem from the unique architectural design of the model, which effectively integrates 3D CNN and LSTM networks to capture spatiotemporal features inherent in basketball actions. Furthermore, the method potentially benefits from advanced data processing techniques and optimization algorithms.

**Table 5:** Performance comparison of different methods on different datasets.

Method	Accuracy on NTURGB + D	Accuracy on the Basketball-Action-Dataset	Accuracy on the B3D Dataset	Remarks
Frame difference	75.3%	69.8%	73.1%	Traditional method
Optical flow	81.4%	76.5%	78.9%	Traditional method
Transformer models	90.1%	87.3%	89.2%	DL method
Reference^37^	86.2%	82.1%	84.3%	DL method
Reference^38^	84.7%	80.5%	82.9%	DL method
Reference^39^	88.5%	84.9%	86.7%	DL method
The proposed method	93.1%	88.5%	90.0%	The proposed method

### Discussion

This section delves into the proposed basketball action recognition method based on 3D CNN and compares it with existing skeleton-based action recognition methods. Existing skeleton-based action recognition methods primarily focus on analyzing human skeleton data. For instance, Yu et al. proposed a Convolutional 3D LSTM method, integrating attention mechanisms into LSTM networks through three-dimensional convolutions. This integration enables memory blocks to effectively learn short-term frame dependencies and long-term relationships^40^. Duan et al. introduced a PoseConv3D skeleton-based action recognition method, potentially enhancing the performance and generalization ability of basketball action recognition models, particularly in multi-person scenarios^41^. Additionally, Lee et al. proposed a Hierarchically Decomposed Graph Convolutional Network architecture, offering promising implications for enhancing the performance and generalization ability of basketball action recognition models, especially in scenarios dealing with skeleton data^42^. In comparison, the proposed basketball action recognition method based on 3D CNN exhibits several innovative aspects. The method proposed here not only focuses on the spatiotemporal features of skeleton data but also directly extracts rich spatiotemporal information from video sequences using 3D CNN, eliminating the need for preprocessing of skeleton joint data. The incorporation of LSTM networks in the proposed method enables the model to capture the long-term dependencies of basketball actions, crucial for comprehending the coherence and complexity of such actions. Through adaptive learning rate adjustment and regularization techniques, the proposed method effectively enhances the generalization ability and robustness of the model during training. Extensively tested on multiple publicly available basketball action datasets, the proposed method validates its recognition performance across various scenarios and conditions. Despite the potentially higher parameter count and computational complexity of 3D CNN models compared to some skeleton-based methods, the proposed approach achieves effective resource utilization while maintaining high accuracy through model optimization and algorithmic enhancements.

In comparison to existing skeleton-based action recognition methods, the proposed approach demonstrates superior recognition accuracy across multiple datasets. Through the application of optimization algorithms, the method exhibits enhanced robustness in identifying basketball actions under varying lighting conditions and complex backgrounds. Furthermore, through cross-dataset testing, the method showcases strong generalization ability, adept at adapting to diverse data distributions and action characteristics.

While the proposed method represents a significant advancement in the realm of basketball action recognition, opportunities for further improvement and optimization persist. Future research endeavors could explore techniques to reduce model parameter count and computational complexity, thereby enhancing the feasibility of real-time applications. Additionally, considering the integration of basketball action recognition with other pertinent tasks such as player identification and tactical analysis through multi-task learning could bolster the overall performance of the model. Moreover, validating the model’s performance on larger and more diverse datasets would provide deeper insights into its generalization capabilities. Furthermore, investigating the integration of video data with other modalities such as audio and text could yield a more comprehensive action recognition solution.

## Conclusion

This research has made significant advancements in enhancing the accurate and automatic recognition of different actions in basketball games by introducing a basketball action recognition technique based on 3D CNNs. The efficacy and robustness of the proposed model are validated through experiments using publicly available basketball action datasets. Throughout the research, basketball action sequences are extracted from these datasets, and comprehensive data preprocessing is conducted. Subsequently, a model based on 3D Convolutional Neural Networks is proposed, which effectively integrates temporal feature modeling using 3D convolution and LSTM networks to capture the spatiotemporal relationships and temporal information of actions. This approach facilitates the automatic learning of the spatiotemporal features of basketball actions. Optimization algorithms such as adaptive learning rate adjustment and regularization are employed during the experiments to further enhance the model’s performance and robustness. The effectiveness and superiority of the model are confirmed through experiments conducted on publicly available basketball action datasets, including NTURGB + D, Basketball-Action-Dataset, and B3D Dataset. A comparison with two common traditional methods based on inter-frame difference and optical flow demonstrates significant performance improvements. Specifically, this model achieves a 15.1% increase in accuracy compared to the inter-frame difference method and a 12.4% improvement compared to the optical flow method. Despite these positive results, the research acknowledges certain limitations, primarily related to the potential for further optimization of basketball action sequence characteristics and spatiotemporal relationships. Moreover, when addressing the challenge of inefficient training due to an excessive number of parameters in 3D CNN models, several strategies or combinations thereof can be employed. Firstly, parameter pruning offers an effective method whereby pruning techniques reduce the number of connection weights in the network, thereby diminishing the parameter count and enhancing the model’s lightweightness. Secondly, model compression techniques, including knowledge distillation and parameter quantization, present viable options to transform large models into smaller, more efficient versions while preserving their performance. Additionally, local connectivity serves as another strategy to reduce parameter count; by introducing local connectivity, the number of connections in each hierarchy can be minimized, thereby reducing the overall parameter count. Parameter sharing is also a common approach to effectively reduce the overall parameter count by sharing some parameters in the network. Finally, redesigning the network structure is also an essential direction, where careful design of the network structure can make it more lightweight while maintaining model performance and reducing the parameter count. Considering these solutions collectively and selecting the appropriate combination according to the specific scenario can effectively enhance the training efficiency of 3D CNN models and mitigate resource and time costs. Furthermore, although this research focuses on the utilization of 3D CNNs for basketball action recognition, it acknowledges that the limitations of this approach may constrain the exploration of potentially more advanced or efficient action recognition methods. With the rapidly evolving fields of computer vision and machine learning, new architectures such as hybrid models, transformers, and even advancements within the CNN framework may offer better performance, efficiency, or insights. Hence, it is recognized that confining the research scope to 3D CNNs may overlook these innovative approaches, thereby limiting contributions to the field. To mitigate this limitation, future research could consider broader investigations and comparisons of other action recognition methods and conduct in-depth contrasts with 3D CNNs. For instance, hybrid models could be explored for basketball action recognition to examine their effectiveness in capturing spatial and temporal features. Additionally, investigating the potential of transformer models in basketball action recognition and how they provide better performance compared to traditional CNNs would be valuable. Such comprehensive studies would yield a deeper understanding of the strengths and weaknesses of different approaches, offering additional insights and recommendations for further advancements in basketball action recognition. Future research endeavors could delve into optimizing model architectures and algorithms to enhance both the accuracy and real-time performance of basketball action recognition systems. These advancements hold promise for enriching the broader domain of sports technology and fostering deeper insights into action recognition within basketball games.

## Data Availability

All data generated or analysed during this study are included in this published article (and its supplementary information files).
